# Low-Cost Point-of-Care Monitoring of ALT and AST Is Promising for Faster Decision Making and Diagnosis of Acute Liver Injury

**DOI:** 10.3390/diagnostics13182967

**Published:** 2023-09-16

**Authors:** Raja Chinnappan, Tanveer Ahmad Mir, Suliman Alsalameh, Tariq Makhzoum, Alaa Alzhrani, Khaled Al-Kattan, Ahmed Yaqinuddin

**Affiliations:** 1 College of Medicine, Alfaisal University, Riyadh 11533, Saudi Arabia; salsalameh@alfaisal.edu (S.A.); tmakhzoum@alfaisal.edu (T.M.); aimalzahrani@kau.edu.sa (A.A.); kkattan@alfaisal.edu (K.A.-K.); 2 Tissue/Organ Bioengineering & BioMEMS Lab, Organ Transplant Centre of Excellence, Transplant Research & Innovation Department, King Faisal Specialist Hospital and Research Centre, Riyadh 11211, Saudi Arabia; 3 Medical Laboratory Technology Department, Faculty of Applied Medical Sciences, King Abdulaziz University, Jeddah 21589, Saudi Arabia

**Keywords:** alanine aminotransferase (ALT), aspartate aminotransferase (AST), paper-based assay, microfluidic devices, lateral flow assay, liver injury

## Abstract

Alanine aminotransferase (ALT) and aspartate aminotransferase (AST) are important liver enzymes in clinical settings. Their levels are known to be elevated in individuals with underlying liver diseases and those consuming hepatotoxic drugs. Serum ALT and AST levels are crucial for diagnosing and assessing liver diseases. Serum ALT is considered the most reliable and specific candidate as a disease biomarker for liver diseases. ALT and AST levels are routinely analyzed in high-risk individuals for the bioanalysis of both liver function and complications associated with drug-induced liver injury. Typically, ALT and AST require blood sampling, serum separation, and testing. Traditional methods require expensive or sophisticated equipment and trained specialists, which is often time-consuming. Therefore, developing countries have limited or no access to these methods. To address the above issues, we hypothesize that low-cost biosensing methods (paper-based assays) can be applied to the analysis of ALT and AST levels in biological fluids. The paper-based biodetection technique can semi-quantitatively measure ALT and AST from capillary finger sticks, and it will pave the way for the development of an inexpensive and rapid alternative method for the early detection and diagnosis of liver diseases. This method is expected to significantly reduce the economic burden and aid routine clinical analysis in both developed and underdeveloped countries. The development of low-cost testing platforms and their diagnostic utility will be extremely beneficial in helping millions of patients with liver disorders.

## 1. Introduction

Blood is one of the most widely used body fluids for monitoring the health status of the liver or diagnosing liver-associated diseases. Hepatologists and gastroenterologists rely heavily on the clinical analysis of liver enzyme levels (serum aminotransferases) to make clinical decisions. In particular, alanine aminotransferase (ALT) and aspartate aminotransferase (AST) levels are crucial for diagnosing and assessing liver diseases. ALT/AST levels are also indicators of overall health status, especially in patients with obesity, metabolic syndrome, and cardiovascular disease [1,2,3]. ALT is primarily aggregated in the cytoplasm of hepatocytes with an intracellular activity of, approximately, 3000-times higher than in serum [4]. When hepatocyte injury occurs, ALT is released from the injured hepatocytes, causing a marked increase in serum ALT activity [4]. This makes serum ALT the most reliable and specific blood-based liver injury or inflammation biomarker for predicting, evaluating, and diagnosing acute and chronic liver disease [5]. Elevated ALT is associated with increased severity of liver diseases, accompanied by symptoms, such as fatigue, anorexia, and pruritus. However, since most patients are asymptomatic at presentation, practitioners do not frequently monitor ALT activity levels, and when they do measure them, slightly elevated levels of ALT are often ignored because of a lack of long-term vision regarding the implications of ALT levels on end-stage liver failure or premature mortality [6]. Non-alcoholic fatty liver diseases (NAFLDs) are one of the principal causes of abnormal ALT levels [7], which are exacerbated by obesity, diabetes, and hyperlipidemia [8]. ALT testing in the early stages of a patient’s disease can facilitate timely treatment as well as help monitor the development of NAFLD and the progression of the disease from simple lipidosis or steatosis to more advanced stages [9]. Traditional diagnostic methods used to measure ALT and AST are based on colorimetry [10], spectrophotometry [11], electrochemistry [12,13], and surface plasmon resonance-based phenomenon [14]. Regardless of the accuracy and specificity of automated or semiautomated analyzers, they require expensive, sophisticated equipment and well-trained professionals. Monitoring the patients’ health using these methods is expensive, especially in developing countries that cannot afford such modern clinical facilities. Developing cheap and simple disposable diagnostic tools independent of peripheral equipment would reduce the economic burden and make them accessible to a large portion of the global population. Therefore, rapid and accurate point-of-care measurement of ALT in the early stages of a patient’s disease is critical for disease management, treatment outcomes, and minimizing economic burden. The successful employment of a non-invasive liver assessment by a paper-based point-of-care biosensing platform would be an ideal device to reduce the time required for sample preparations and laboratory establishment. This method is qualitative or semi-quotative; however, the detection limit is within the recommended clinical range. Therefore, this device can be applied for point-of-care (do-it-yourself) application. We hypothesize that by employing a point-of-care strategy, patient samples could be collected immediately at the sampling point and analyzed for disease detection and screening without the need to engage highly specialized equipment and personnel to interpret the data. This cost-effective technology could make a significant contribution to the diagnosis and treatment of liver-related health disorders and the control of disease progression.

It is worth noting that in developing countries, conventional pathology laboratories are expensive for diagnosing diseases and monitoring patient health. Therefore, there is great anticipation from researchers to develop simple, cheap, and easy-to-use point-of-care (POC) diagnostic tools to diagnose and monitor patients’ health status. Several research groups have reported strategies for POC diagnosis by detecting metabolites and proteins using paper, thread, and magnetic levitation [15,16,17,18]. In addition, inexpensive colorimetric enzyme-linked immunosorbent assays (ELISAs) using paper have also been developed to detect antibodies in biological fluids [19]. Although many papers have been published on the development of POC diagnostics, most of them have not yet been put into practice. Therefore, further developments and improvements are needed in realizing paper-based technologies for commercial applications. 

## 2. Hypothesis: Robust and Rapid Method for ALT Diagnosis at the Point-of-Care Testing for Monitoring Liver Damage and Function

The design and development of low-cost, simple paper-based point-of-care testing devices to monitor health-related parameters (analyte) from blood or urine for disease diagnosis in resource-limited settings have attracted public health authorities and medical practitioners in recent years [20,21]. Here, we propose that an efficient and extremely low-cost disposable assay device can be a valuable tool for the detection of ALT from blood. POC testing can reduce hospital visits and healthcare costs and achieve patient satisfaction by visualizing results with the naked eye and reaching the end-user easily. Paper-strip-based tests for pregnancy, diabetes, drug abuse, and pathogen biomarkers are already commercially available. In these tests, the sample flows along the paper strip through capillary action, and the color of the detection zone changes as the analyte binds to the reagent zone.

Currently, ALT levels are frequently evaluated using spectrophotometry, chemiluminescence, colorimetric, chromatography, enzymatic kinetic reaction, and SDS-PAGE-based methods [22]. In most cases, the ALT/AST levels are measured from whole blood, plasma, or serum. The basic principle of colorimetric and spectrophotometric assays is based on enzymatic reactions with substrates, producing products that interact differently with light compared to the reactants. Lippi and Guidi reported an ultramicro-colorimetric method for the detection of ALT and AST. This method utilizes the reduction of dehydrogenated glutamate to its disodium salt, followed by the measurement of produced compounds in color, and forms an absorption peak at about 520 nm [10]. Aptamers are nucleic acid having 40 to 100 bases serving as an emerging recognition receptor, which replace the antibodies for the fabrication of various low-cost biomedical diagnostics biosensors. They are more advantageous compared to antibodies, such as low production cost, high thermal stability, and easy chemical modification. The single-strand oligonucleotide aptamers undergo unique conformation change in the presence of target analytes and bind with high affinity and specificity [23,24]. In vitro screening of aptamers by systematic evolution of ligands by exponential enrichment (SELEX) methods is commonly used against the target molecules. Numerous aptamers have been selected against different kinds of targets, ranging from small metal ion to large whole cell with high affinity and specificity [24,25,26,27]. Aptamers are ideal candidates as a recognition element in biosensor development that bind the target with high affinity followed by translation and measurable signal via a transducer. The flexibility of the aptamer will be utilized for the fabrication of point-of-care testing with an affordable, sensitive, specific, user-friendly, rapid and robust, equipment-free, and deliverable (ASSURED) device. Paper-based ALT and AST detection uses anti-ALT and anti-AST aptamers as recognition elements, in which the partial complementary and complementary aptamer sequences are immobilized at the test line and control lines, respectively. In the presence of ALT- and AST-positive samples, the target molecules complexed with the gold nanoparticle-labelled anti-ALT/anti-AST aptamers in the conjugate pads and moved towards the test lines. The test lines having the partial complementary or secondary aptamer, based on the aptamer-target binding ration, produce the color on the test line. The control lines have a long complementary capturing agent, which captures the excess aptamers, validating the performance of the device [28]. Similarly, an aptamer-based lateral flow assay was developed for the semiquantitative detection of the new anticoagulant drug Dabigatran etexilate (DBG) using aptamer-conjugated gold nanoparticles [29]. The chemiluminescence method is also widely used to detect ALT and AST because of its high sensitivity, low background noise, and ability to measure a wide range of concentrations. In the enzymatic reaction of ALT, L-alanine is broken down into pyruvate and L-glutamate, and pyruvate oxidase converts pyruvate to hydrogen peroxide. Luminol then reacts with hydrogen peroxide to produce chemiluminescence [30]. Chromatographic detection of ALT and AST has also been achieved by combining gas–liquid chromatography with an electron capture detector. This method allows for the detection of sub-micromolar levels while identifying all components in the reaction mixture [31]. Alternatively, a highly sensitive fluorometric method has been developed to detect ALT/AST enzymes, both in solution and through a solid-surface method by tracking the diminishing fluorescence of NADH at 455 nm (λ_ex_ = 365 nm) and correlating it with the enzyme concentration in serum [32]. Recently, electrochemical methods have attracted significant attention as well. Target analytes are detected using various enzyme-modified electrodes, such as pyruvate oxidase/glutamate oxidase. Using the two-electrode method, pyruvate oxidase can be immobilized on one electrode, and co-immobilization of oxaloacetate decarboxylase and pyruvate oxidase on another electrode can be accomplished. This way, both ALT and AST activities can be detected simultaneously within 4 min [33]. Although both methods can reliably detect ALT and AST with high sensitivity, they have limitations. The spectrophotometric method needs a spectrophotometer and other accessories. Moreover, the results often need to be more accurate for samples with icteric-, lipemic-, or homolysis-derived byproducts. Additionally, the presence of inhibitors, such as superoxide dismutase or N-nitro-L-arginine methyl ester hydrochloride, in the sample impairs the response pattern. For chromatography, the main drawback is the possible presence of biomolecules in the sample that exhibit the same chromatographic behavior as the target molecule. Fluorescence, on the other hand, is highly sensitive to the local environment (pH, ionic strength, solvent polarity, temperature, quenching by the sample matrix, etc.) and requires careful handling to obtain reliable results. Electrochemical measurements can be hampered by nonspecific adsorption of biomolecules on the electrode surface or low protein loading on the electrode surface. Therefore, conventional ALT measurement methods require blood to be collected by venipuncture and centrifuged to separate the serum. Serum samples are then tested by trained specialists using large automated or sophisticated equipment. These processes are expensive and require highly trained personnel. In addition, medical monitoring of patients in distant areas is rarely conducted because the operation and maintenance of sophisticated equipment are impractical and a major barrier in remote centers.

Although there are many ALT test kits available commercially, they still need laboratory settings for solution preparation and pipetting and may require readout instruments. In this hypothesis, we propose the use of a simple point-of-care paper-based biosensing platform for liver function testing services. 

## 3. Evaluation of Hypothesis: Point-of-Care Test for Diagnosis and Monitoring Liver Function Using ALT and AST Enzymes

**Figure 1:**
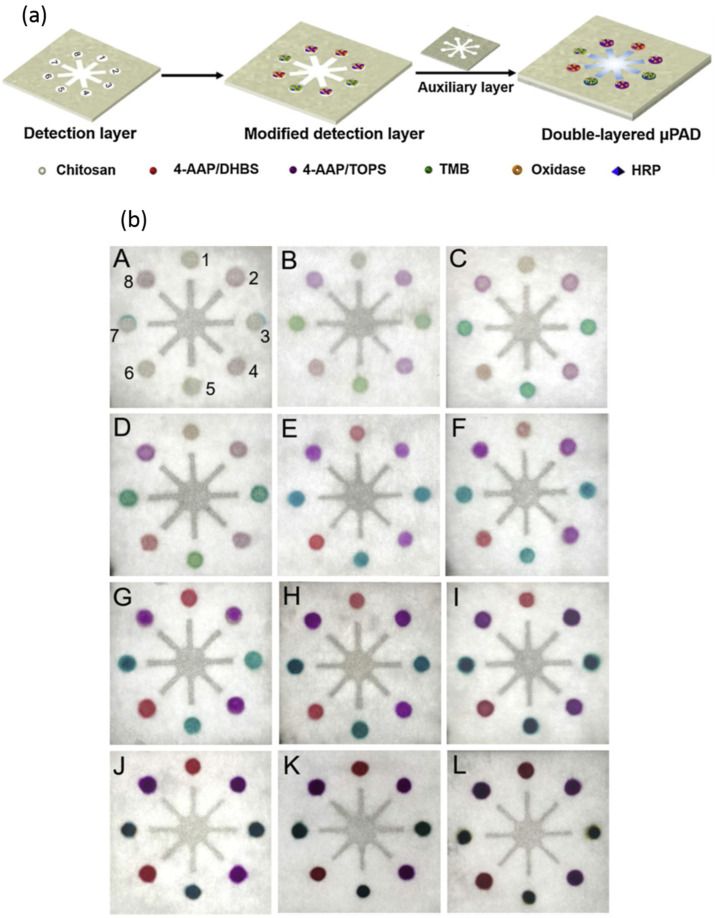

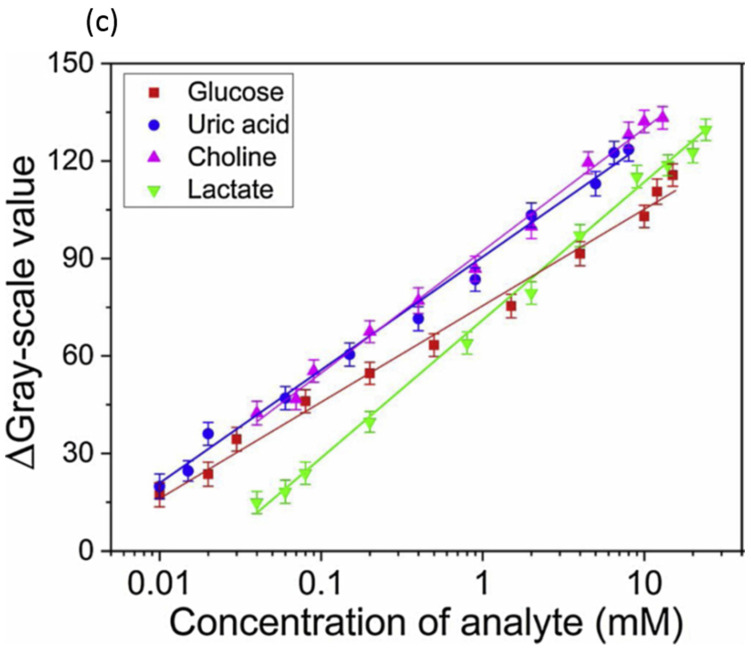
(**a**). Pictorial representation of the double-layered microfluidic device with multiple indicators for the multiplex detection of four analytes simultaneously. (**b**) The colored pictures of the detection layer of the μPAD in the presence of blank buffer. (A) Variable concentrations of glucose, uric acid, choline, and lactate. The concentrations of glucose, uric acid, choline, and lactate were 0.01, 0.01, 0.04, 0.04 mM (B); 0.02, 0.015, 0.07, 0.06 mM (C); 0.03, 0.02, 0.09, 0.08 mM (D); 0.08, 0.06, 0.2, 0.2 mM (E); 0.2, 0.15, 0.4, 0.8 mM (F); 0.5, 0.4, 0.9, 2 mM (G); 1.5, 0.9, 2, 4 mM (H); 4, 2, 4.5, 9 mM (I); 10, 5, 10, 14 mM (J), 12, 6.5, 11, 20 mM (K), 15, 8, 13, 24 mM (L), respectively. The detection zones were numbered 1 to 8. Positions 1 and 2 are glucose detection zones; positions 3 and 4 are uric acid detection zones; positions 5 and 6 are choline detection zones; positions 7 and 8 are lactate detection zones. The positions are the same for all the detection zones from A to I. (**c**) The calibration plot of the concentration of glucose, uric acid, choline, and lactate against the color intensity values obtained from the device. Reprinted with permission from ref. [35].

Paper-based microfluidic analytical devices have attracted much interest as a promising platform for point-of-care applications in medical, environmental, food safety, and security fields. Various types of paper-based microfluidic devices for clinical applications have been developed. These devices employ simple patterning methods that allow for the manipulation of sample flow and execution of chemical and biochemical reactions without the need for external pumps or power devices. The basic principle of this device is based on the oxidation of a colorimetric probe by hydrogen peroxide produced from the analyte-specific oxidases. Tian et al. developed an optical-based method for the detection of ALT using the peroxidase (POD)-like activity of B-doped core–shell Fe@BC nanozyme. ALT converts L-alanine and α-ketoglutarate into L-glutamate, and the oxidation of L-glutamate by glutamate oxidase produces H_2_O_2_, which further oxidizes 3,3′,5,5′-tetramethylbenzidine (TMB) to oxTMB by the POD-like activity of Fe@BC nanozyme, resulting in a blue-colored compound. The assay was measured in the dynamic range of 10 to 1000 U/L with a detection limit of 4 U/L [34]. Paper-based colorimetric assays for the detection of ALT and AST from various reports are summarized in Table 1. Li et al. developed a double-layered microfluidic paper-based device for the simultaneous detection of clinically important biomolecules, such as glucose, uric acid, lactate, and choline in a mixture, in which multiple colorimetric indicators were introduced at the detection spots. The device is an integration of the top detection layer and the bottom auxiliary layer. The top detection layer had different kinds of colorimetric reagents and respective oxidases (glucose oxidase, uricase, lactate oxidase, or choline oxidase) and HRP (Figure 1a). The colorimetric agents on the detection spots were oxidized using hydrogen peroxide (H_2_O_2_) generated from the reaction between the corresponding oxidases and the substrate, which produce different colored products with the HRP as a catalyst (Figure 1b). A linear relationship between the concentration of the analytes and the color intensities was obtained. The device was used to measure glucose, uric acid, lactate, and choline in the linear range of 0.01–10.0 mmol/L, 0.01–5.0 mmol/L, 0.04–10.0 mmol/L, and 0.04–24.0 mmol/L, respectively. The numbers 1 and 2 were used for glucose detection that produce red and purple, numbers 3 and 4 were used for uric acid detection that produce blue-green and purple color, numbers 4 and 6 were used for choline detection that produce blue-green and red color and the numbers 7 and 8 were used for lactate detection that produce blue-green and purple color respectively. (Figure 1c). The detection limits were 0.003, 0.005, 0.03, and 0.01 mM for glucose, uric acid, lactate, and choline, respectively [35]. In another microfluidic paper-based assay, Shariati et al. demonstrated the sensitive detection of cysteine and homocysteine using 1,5-diphenylcarbazide (DPC)-capped silver nanoparticles. Diphenylcarbazide has two amino groups that interact with the surface of silver nanoparticles through hydrogen bonding and electrostatic interactions (1,5-diphenylcarbazide). In general, DPC is used as an indicator for iron. It is also used for the colorimetric detection of Cr (VI). The detection of the analytes is based on the colorimetric reaction of modified silver nanoparticles with the reagents. The modified silver nanoparticles in the test zone changed their color in the presence of cysteine and homocysteine targets. RGB color model and ImageJ software were used for the quantification of the analytes. Under optimized conditions, this device detects cysteine in a range of 0.2–20 mM with a detection limit of 0.16 mM. Homocysteine was measured in a range of 0.5–20.0 mM with an LOD of 0.25 mM. This device could be used for urine analysis with point-of-care applications [36]. 

A recent review by Noviana et al. detailed the design, production, and application strategies of one such device [37]. Based on this principle, Whiteside and his team reported an affordable paper-based analytical device for clinical applications [38,39,40]. Patients with liver diseases can use these devices to test their ALT/AST levels using a drop of blood through a finger puncher. In another study, Pollock et al. demonstrated a paper-based, multiplexed, microfluidic device that provides accurate visual readings of transaminase enzymes, ALT, and AST within minutes using fingerstick whole-blood specimen [41]. This paper-based device is both compact and can perform one or two tests in parallel without cross-reactivity. To ensure accuracy and ease of use, the device has been optimized to read ALT and AST ranges in colors corresponding to values below the upper limit (<3), upper normal (3–5), and above upper normal (>5). These values are currently used to determine clinical management in TB and HIV treatment guidelines in the USA [42]. 

**Figure 2:**
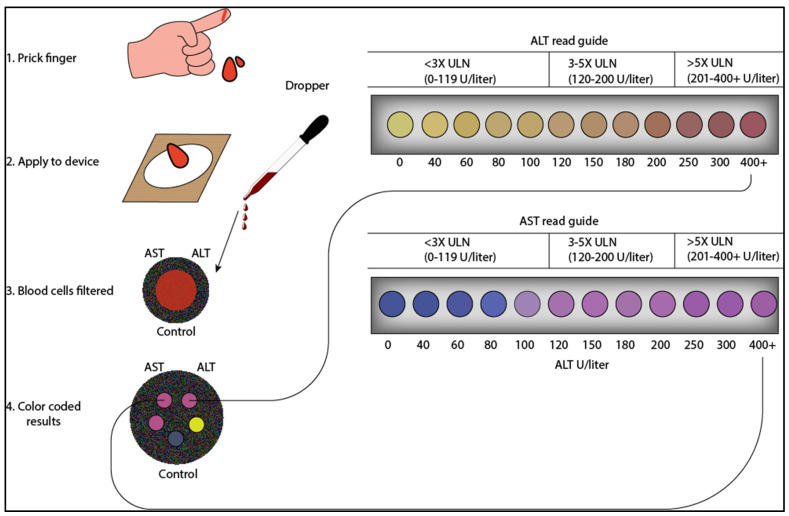
Paper-based assay developed for semi-quantitative analysis of ALT and AST from whole blood obtained from venipuncture. In this device, the red and white blood cells are filtered by a plasma separation membrane. The plasma wicks to five different ALT/AST test zones. After 15 min, the color developed on the test zones is compared with the color read guide to estimate the ALT and AST levels (figure designed from biorenders.com).

The transaminase paper-based device is made from a layer of patterned paper; using a wax-based printer, a heat source introduces microfluidic and hydrophilic paths within the paper, which direct the fluids to the detection zones. Within this postage-stamp-sized device (20 × 20 × 0.5 mm, two tests (each with a detection zone) are performed: one measuring ALT and the other measuring AST. Additionally, the device has a control zone to check functionality. Each detection zone is set with a reagent that changes color in reaction with a specific enzyme. ALT is measured based on the conversion of L-alanine and alpha-ketoglutarate to pyruvate and L-glutamic acid. Pyruvate oxidase oxidizes pyruvic acid, liberating hydrogen peroxide. Subsequently, 4-aminoantipyrine and N,N-dimethylaminobenzoic acid undergo a coupling reaction in the presence of horseradish peroxidase and hydrogen peroxide to produce red-colored dye. Similarly, AST assay chemistry is used to detect AST in the sample. AST converts cysteine sulfinic acid and alpha-ketoglutaric acid to L-glutamic acid and beta-sulfinyl pyruvate. In the presence of water, beta-sulfinyl pyruvate generates SO_2_^-^ which reacts with blue-colored methyl green and produces a colorless compound. The device consists of two identical sheets of patterned paper with a plasma separation membrane laminated with a polyester film that prevents evaporation and protects from the environment. When a small volume (30 µL) of whole blood/serum is applied to the plasma separation membrane, blood cells are blocked by the membrane, leaving plasma to migrate from the first to the second layer. The plasma then interacts with dried reagents within the detection zone and develops a color. A colorimetric read guide can be employed for interpretation and semi-quantitative quantification because of the linear relationship between observed color density and the clinical range of ALT and AST. As shown in Figure 2, the interpretation based on the reading guide roughly aligns the results to one of three bins (<3 × ULN (0 to 119 U/liter), 3 to 5 × ULN (120 to 200 U/liter), or >5 × ULN (>200 U/liter)). Finally, a control zone checks the performance of the device. 

The paper-based assay results are compared with the gold-standard automated serum transaminase results to validate the sensitivity and specificity. We hypothesize, based on similar devices, that the overall accuracy of this device will be more than 90% for both ALT and AST enzymes. 

Regardless, this device semi-quantitatively detects the levels of transaminase enzymes from minute quantities of the blood, estimating liver function within 15 min, which is less than the conventional analysis time (>1 h). 

Since this device does not require any external equipment, patients can check their ALT levels with this paper-based instrument at home with blood collected with a finger puncher. As a result, patients can better decide whether or not to seek medical attention based on the color intensity of their blood. No such paper-based analyzer for ALT/AST measurement is commercially available to date. This is due to the asymptomatic nature of liver disease and its increasing prevalence, especially in remote and resource-limited settings, where there is an urgent need for the commercial availability of paper-based devices to monitor liver function.

## 4. Consequences of the Hypothesis and Discussion

**Figure 3:**
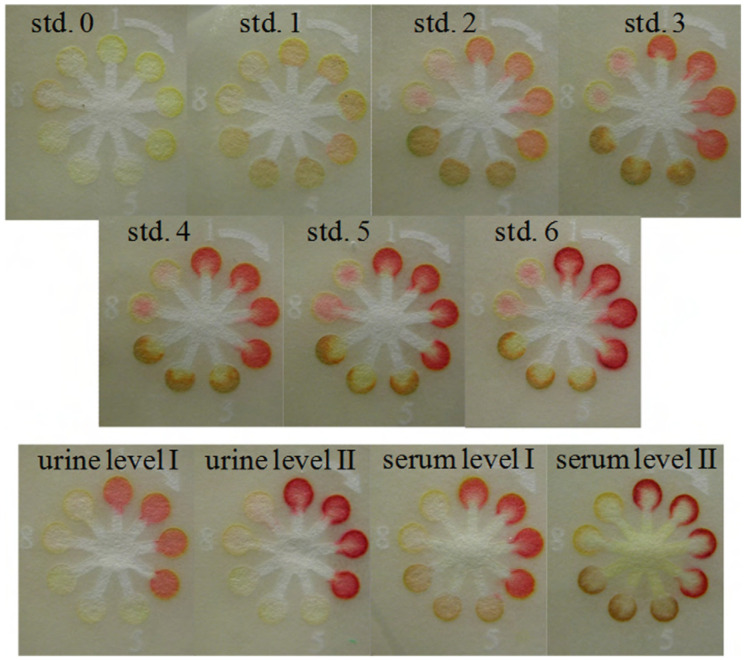
Colorimetric paper-based microfluidic device sensors for the simultaneous semi-quantitative analysis of glucose, lactate, and uric acid. Position numbers 1 to 4 are glucose test zones using Y + AB as an indicator; position numbers 5 to 7 are lactate test zones using Y + OD as an indicator, position numbers 8 and 9 are uric acid test zones using AB indicator. The abbreviation of the indicators: 4-aminoantipyrine (AAP) and 3,5-dichloro-2-hydroxy-benzenesulfonic acid (DHBS) in the mole ratio of 1:2 (AB), o-dianisidine dihydrochloride (OD), potassium iodide (KI), acid yellow 34 (Y), and acid black 1 (B). Reprinted with permission from ref. [43].

The results of ALT and AST colorimetric assay are quantified by comparing the color density produced by the unknown sample to known standards. Color matching using the naked eye may be complicated by many factors, such as color perception, background lighting, and color differences between the dry-printed guide and the wet-paper device. To bridge the gap, quantitative analysis of the diagnostic test was performed using additional equipment, high-resolution cameras, and scanners. To circumvent these obstacles, multiple indicators for a single analyte generate multiple colors for better visual discrimination. In one study, Dungchai et al. demonstrated semi-quantitative measurement of glucose, lactate, and uric acid in a paper-based microfluidic assay using multiple indicators. In this approach, each indicator reacted to different concentrations of peroxides and exhibited a variable range of colors for the accurate analysis of analytes. The single indicator test had an accuracy of 70%, whereas multiple indicators were over 90% accurate [43]. The combination of more than one indicator with known concentrations was placed in the test zone. Standard color intensities were developed for the specific concentrations of glucose, lactate, and uric acid, as shown in Figure 3. The color intensities of the unknown urine and plasma samples were compared with the standards for the semi-quantitative analysis (Figure 3). Therefore, we suggest a multiple-indicator approach for ALT and AST to further improve the accuracy of the results.

## 5. Conclusions and Future Prospects

In conclusion, we hypothesize that paper-based assays for ALT and AST testing are rapid, extremely inexpensive, and user-friendly, and they can be used to self-monitor liver function at home and with simple fingerstick specimens. This would be more suitable for regions lacking proper healthcare infrastructure. The test would require a commercially available ALT/AST test stick, a mini finger-punch needle, and a capillary tube. This assay is extremely inexpensive compared to conventional methods and allows for the adequate follow-up of the ALT/AST range through qualitative/semi-quantitative measurements. Immediate implementation of a paper-based colorimetric assay would allow for timely treatment and prevent advanced stages of liver damage. In addition, paper-based aptasensors are considered as a potential device for future point-of-care applications. Though there are some practical limitations in the field/in vivo applications, such as nuclease digestion and rapid renal filtrations. Fast-developing modern technology would overcome these limitations in the future.

**Table 1:** Paper-based colorimetric assay developed for ALT and AST detection.

Analyte	Analytical Method	Materials Used	Detection Range	LOD	Reference
ALT/AST	Hydrogel-Colorimetric	Au-decorated CoAl-layered double oxide (Au/LDO)	-	15 U/L (ALT) 10 U/L (AST)	[44]
ALT	Paper-based Colorimetry	2,4-dinitrophenyl hydrazine	20–140 U/L	4.12 U/L	[45]
ALT	Paper-based Colorimetry	ALT assay Chemistry *	40–200 U/L	53 U/L	[41]
AST	Paper-based Colorimetry	AST assay Chemistry **	40–200	84 U/L	[41]
ALT	Paper-based Colorimetry	Inkjet Printer	5.38–8.61	N/A	[21]
AST	Paper-based Colorimetry	Inkjet Printer	5.4–91.2	N/A	[21]
ALT	Colorimetry	B-doped core–shell Fe@BC nanozyme/L-alanine and α-ketoglutarate	10–1000	4 U/L	[34]

* ALT assay chemistry: ALT converts L-alanine to pyruvate, subsequently oxidation of pyruvate by pyruvate oxidase. The liberated hydrogen peroxide was used to produce a red-colored compound by coupling 4-amino antipyrine and N,N-dimethylaminobenzoic acid using horseradish peroxidase. ** AST Assay Chemistry: sulfonation of methyl green by AST leads to a visual color change from blue to pink. The product is colorless, the pink (Rhodamine B) background color appeared.

## Data Availability

Not applicable.
